# Characterizing an angle of cannula insertion for Lumbar Medial Branch Radiofrequency Neurotomy: A retrospective observational study

**DOI:** 10.1016/j.inpm.2022.100071

**Published:** 2022-02-17

**Authors:** Ajay K. Patel, Jason L. Chang, Paul R. Haffey, Ojas Mainkar, Amitabh Gulati

**Affiliations:** a Department of Rehabilitation & Regenerative Medicine, NewYork-Presbyterian Hospital- University Hospital of Columbia and Cornell, New York, NY, USA; b Department of Rehabilitation & Regenerative Medicine, NewYork-Presbyterian Hospital-Columbia University Medical Center, New York, NY, USA; c Department of Anesthesiology, NewYork-Presbyterian Hospital-Weill Cornell Medicine, New York, NY, USA; d Department of Anesthesiology and Critical Care, Memorial Sloan Kettering Cancer Center, New York, NY, USA

**Keywords:** Chronic pain, Lumbar pain, Radiofrequency neurotomy, Zygapophyseal joint, Medial branches, Cannula placement

## Abstract

**Objective:**

Evidence characterizing a starting angle of radiofrequency (RF) cannula insertion during radiofrequency neurotomy is lacking. Using computerized tomography (CT), this retrospective observational study attempts to establish a starting angle for RF cannula placement parallel to the transverse process (TP) at the junction of the superior articular process (SAP) near the targeted medial branch.

**Methods:**

This retrospective observational study utilized lumbar spine CT scans performed on adult cancer patients from January 2016 to May 2021 ​at a single center. No significant lumbar pathology was present on the included CT studies. For each patient, medial branches were assumed to lie at the junction of the right and left TP and SAP at each lumbar level. The angle of insertion from each segment’s “squared” superior end plate needed for RF cannula placement parallel to the surface of the TP next to the SAP was calculated.

**Results:**

Images obtained from fifty patients were analyzed. Mean angle of insertion for L1 was 20.15 ​± ​1.82°, L2 was 20.95 ​± ​2.07°, L3 was 25.54 ​± ​1.76°, L4 was 31.01 ​± ​1.83°, and L5 was 40.74 ​± ​1.86°.

**Conclusion:**

This study demonstrates variations in inserting angle for RF cannula placement parallel to the surface of the transverse process at each lumbar level. To our knowledge, there are no studies in the current literature that have described an entry angle for RF cannula positioning parallel to lumbar medial branches using CT images.

## 1 Introduction

Lumbar facet joint arthropathy is an established source of low back pain [[1], [2], [3], [4], [5]]. Each lumbar facet joint is innervated by two medial branches of the primary dorsal rami of the corresponding spinal nerve [3]. Lumbar Medial Branch Radiofrequency Neurotomy (LMBRFN) is a common treatment for individuals whose pain has been refractory to other treatments. The goal of LMBRFN is to provide patients with long-term pain relief. However, there is conflicting evidence regarding its efficacy due to the unique anatomy of the medial branch [2,3,5,6].

Each medial branch nerve arises from its dorsal ramus and courses along the SAP posterior to the foramen [4]. Beneath the mamillo-accessory ligament, the nerve traverses medially to supply the multifidus muscle [4]. Early cadaveric studies found that each medial branch resides within the multifidus muscle and only those muscle fibers attaching to the vertebra of the same number as the nerve were innervated [7]. In these early investigations, the target for RF cannula placement to isolate the medial branch was along the dorsal aspect of the transverse process caudal to the medial end of the superior edge of the transverse process [7].

Current techniques target the lateral aspect of the base of the SAP in an attempt to place the RF cannula parallel to the medial branch [8]. This is facilitated through the utilization of declined and oblique rotation of the fluoroscope [8]. Utilizing this parallel orientation yielded superior outcomes based on pain scores and patient self-reported benefit [[8], [9], [10]]. However, evidence demonstrating a standardized angle of RF cannula insertion is lacking.

Using computerized tomography (CT) radiography, this retrospective observational study sought to determine calculated angles of RF cannulae insertion at each lumbar level in order to improve accuracy in parallel placement of the cannulae along targeted medial branches in patients undergoing radiofrequency neurotomy for the treatment of axial low back pain.

## 2 Methods

### 2.1 Data sources

PubMed, PubMed Central, and Google Scholar were the primary sources for literature review for this manuscript.

### 2.2 Study design

This study was approved by the Institutional Review Board of Memorial Sloan Kettering Cancer Center and was supported by the Department of Anesthesiology and Critical Care (NIH Core Grant P30). The study was granted waiver of informed consent because it evaluated existing records, was not greater than minimal risk, and was deemed to be Health Insurance Portability and Accountability Act compliant because safeguards were in place to protect the personal health information of the subjects. This single center, retrospective observational study utilized whole spine lumbar CT scans performed on adult cancer patients at Memorial Sloan Kettering Cancer Center from January 2016 and May 2021.

### 2.3 Study population

An existing database included 160 patients with CT spine radiography. For each patient, sociodemographic information including sex, age, primary cancer diagnosis, height, weight, and BMI was extracted from the medical records. See Table 1. Records were reviewed to identify patients with total or lumbar CT spine imaging, and only one imaging series was selected for each qualifying patient. Patients with radiographs showing lumbar spine instrumentation, infection, primary or metastatic disease, compression fractures, kyphoplasty and laminectomies were excluded. Of the original 160 patients reviewed, fifty patients fit the inclusion criteria (19 male and 31 female).

**Table 1:** Patient demographics.

	Study Population
Sample size, n	50
Sociodemographic Characteristics	
Mean age (SD) in years	67.4 (12.4)
Sex, n (%)	
Male	19 (38%)
Female	31 (62%)
Mean height (SD) in centimeters	164.6 (10.2)
Mean weight (SD) in kilograms	74.0 (19.9)
Mean Body Mass Index (SD) in kilograms/meters^2^	26.6 (5.5)

Abbreviations: SD, standard deviation.

### 2.4 Data collection

Each patient’s imaging was reviewed separately by two independent reviewers (AP and JC). Images were accessed using the Picture Archiving and Communication Software (PACS). For each patient, lumbar sagittal images were reviewed to identify the junction of the superior articulating process (SAP) and the transverse process (TP) of each lumbar level, L1-L5, on both the left and right sides.

**Fig. 1:**
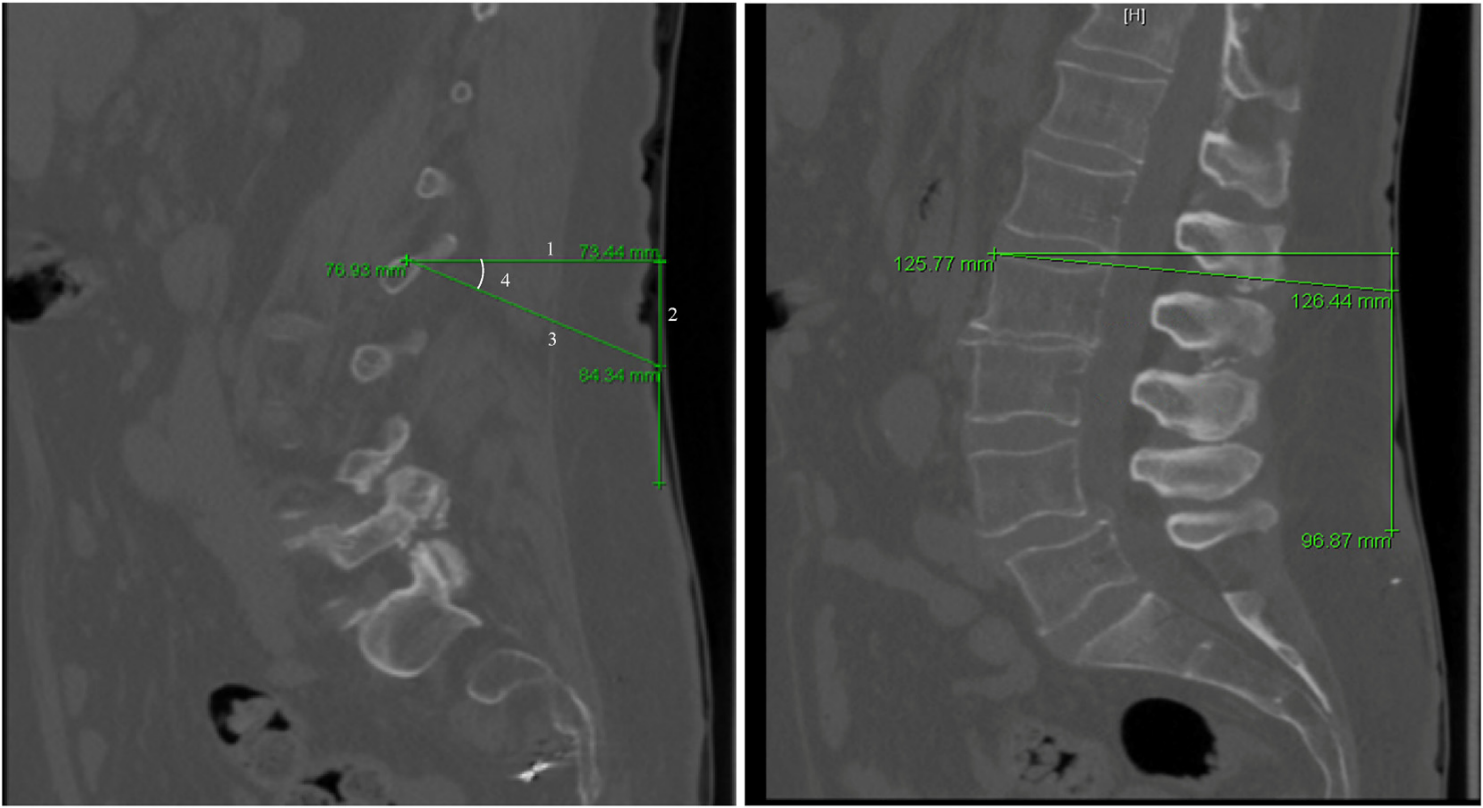
Data Collection Method An example of how each measurement was collected. A. 1) A line was drawn perpendicular to the skin from the junction of the TP and SAP. 2) A line was drawn perpendicular to line 1. 3) A line was drawn oblique until it intersected with line 2. 4) Angle of insertion was calculated. B. A similar procedure was followed to calculate the angle needed to “square” the vertebral body.

Using the annotation tools provided in PACS, a perpendicular line was drawn from the junction where the medial branch is targeted during radiofrequency neurotomy to the skin. A second line perpendicular to the first line and directed along the skin caudally. Lastly, an oblique line was drawn from the junction, to connect to the second line (Fig. 1). Using the length of the oblique and perpendicular lines, along with the cosine formula, the precise angle needed to target the medial branch was determined. The angle needed to “square” the superior end plate at each level followed a similar process and utilized the two angles to obtain the optimal cannula insertion angle relative to the “squared” end plate. This procedure was repeated at each of the five lumbar levels bilaterally one for each of the fifty patients. Any discrepancies beyond 5° were assessed by PH and AG and repeat measurements were made for additional data points. Of note, this process was performed for only the angle needed to place a cannula parallel to the TP and SAP junction.

### 2.5 Statistical analysis

Patient information and demographics were recorded in Microsoft Excel 2016. For each lumbar level and side, the radiography date and image number utilized were recorded. As described in Fig. 1, the distance perpendicular to the skin from the superior end plate and junction (a), the distance of the oblique line to the perpendicular caudal line from the superior end plate and junction (b), and the calculated angle of RF cannula insertion relative to a starting position perpendicular to a “squared” superior end plate using the cosine formula: ​= ​ACOS((a)/(b))∗180/PI were determined. The angle of insertion was corrected by addition or subtraction to account for the angle needed to “square” the superior end plate on imaging. The mean formula to determine the mean age, weight, height, and BMI of the study population was then performed. A z score of 1.96 was used to represent a 95% confidence interval. Using the mean, standard deviation (SD), and a confidence coefficient of 1.96 the margin of error (MOE) and confidence interval (CI) were determined for our calculated angles L1-L5. Our data was then further stratified our data based on laterality (left vs. right) for each lumbar level, sex, age, and BMI.

### 2.6 Interobserver reliability

Interobserver reliability was compared for the final angle of insertion. The percent concordance was determined for each level and laterality. A five-degree acceptable error was utilized when calculating the concordance between the two data collectors. An inherent cannula error of around 2–3° was calculated when entering the skin while being coaxial with the fluoroscopic beam and another 2–3° error when adjusting the fluoroscopic angles as fluoroscopic angle labels are only in 5° or 15° increments. While these are estimates, predetermination was done with a protractor on acceptable images while a cannula was coaxial and the change in angle of the fluoroscope to before coaxial cannula was evident as not coaxial. Based on this five-degree threshold, the number of measurements were tabulated and divided it by the total number of measurements for each respective lumbar level and laterality. A Cohen’s kappa statistic could not be calculated for this study as there is no absolute true value that our measured angles could be compared to. Fleiss’ kappa could not be used because there were only two raters.

## 3 Results

**Fig. 2:**
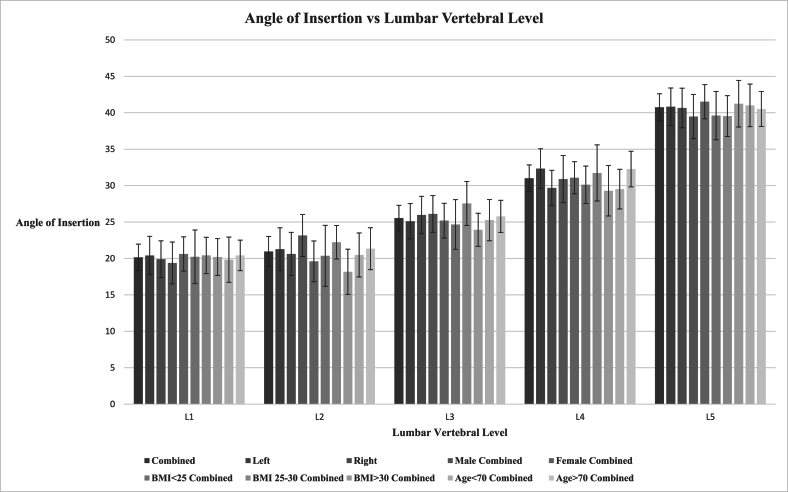
Angle of Insertion vs. Lumbar Vertebral Level.

Fifty total patients were analyzed, consisting of 19 male and 31 female. Mean age of the studied population was sixty-seven years old, weight 74 ​kg, height 165 ​cm, and BMI 26.6. Mean angle of insertion for L1 was 20.15 ​± ​1.82° 95% CI [18.33, 21.96], L2 was 20.95 ​± ​2.07° 95% CI [18.87, 23.02], L3 was 25.54 ​± ​1.76° 95% CI [23.78, 27.30], L4 was 31.01 ​± ​1.83° 95% CI [29.18, 32.84], and L5 was 40.74 ​± ​1.86° 95% CI [38.88, 42.60]. When separated based on laterality, means were rather similar between right and left and the confidence intervals overlapped. Stratifying the data based on sex, BMI (normal weight, overweight, and obese), and age (younger than 70 years old versus older than 70 years old), there were no statistically significant differences in terms of the angle for the lumbar segments. See Fig. 2.

### 3.1 Interobserver reliability

Given as a mean, interobserver agreement for right-sided observations were as follows: L1-86%, L2-86%, L3-98%, L4-94%, L5-82%. As a mean, interobserver agreement for left-sided observations were as follows: L1-90%, L2-94%, L3-98%, L4-94%, L5-86%. Concordance for each level regardless of laterality were as follows: L1-88%, L2-90%, L3-96%, L4-89%, and L5-84%.

## 4 Discussion

The major finding of the retrospective observational study was that the approximate mean angle of insertion for LMBRFN at each lumbar level were as follows: L1 20°, L2 20°, L3 25°, L4 30°, and L5 40° caudally relative to a starting a starting position perpendicular to the superior vertebral end plate. The lower lumbar levels require larger angles of insertion due to natural lumbar lordosis requiring more of a cephalad tilt to “square” the end plates. The differences in means of angle insertion at the lower lumbar segments elucidates that there are potentially different optimal angles of approach at different lumbar segments. Despite starting out with an existing database of 160 lower back pain patients with CT spine radiography, we had to narrow down to fifty patients after we excluded patients with lumbar spine instrumentation, infection, primary or metastatic disease, compression fractures, kyphoplasty and laminectomies for the results to be more applicable to the general population. Future studies can include large sample sizes to improve the confidence intervals.

Radiofrequency efficacy is dependent on positioning of the active electrode tip along the usual course of a nerve [11]. Electrical conductivity differences between surrounding lumbar soft tissue and bone alters the electrical current density and resulting RF lesion shape [11]. When placed in soft tissue alone, the lesion sizes are smaller compared to when placed near bone and soft tissue [11]. Studies have shown that neurotomy is most effective when an electrode is placed parallel to a bone to optimize surface lesion size [11]. As discussed by Loh et al., the parallel placement technique involves placing curved electrodes tangentially along the course of the nerve, allowing longitudinal contact between the cannula and the nerve [8]. Parallel placement of radiofrequency cannula(e) against the medial branch nerves demonstrates superior outcomes in magnitude and duration of relief [8]. Furthermore, a recent consensus practice guidelines by Cohen et al. reported a high certainty of moderate benefit for near-parallel placement of electrodes to increase the likelihood of medial branch RFA and recommended that physicians provide this service in their clinical practice [10]. A larger surface area of neurotomy on the TP and SAP junction may be achieved utilizing our angle measurements, which may increase the likelihood of ablating the medial branch nerves. Given our data, one option to further improve placement of RF cannula, a CT lumbar spine evaluation may be helpful. MRI of the lumbar spine can potentially be a reasonable substitute, especially given the reduced radiation exposure. At our institution, patients had lumbar spine CT images already available for study. Having CT images in the community may not be commonplace, thus the calculated means for angle insertion may be a reasonable starting point for cannula placement.

Furthermore, an AP approach to the TP SAP junction was described, which requires visualizing the SAP and placing a cannula lateral to the SAP. An additional oblique angle may further increase the likelihood of lesioning the nerve on the bony surface, however, an angle to achieve this was not feasible given the absence of oblique images of the spine [12].

**Fig. 3:**
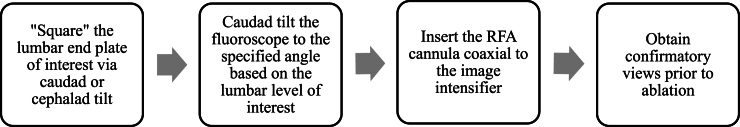
RF procedural protocol using characterized angles of insertion.

The primary principles for effective outcomes following the LMBRFN procedure consist of parallel and proximate placements of lesioning electrodes next to targeted medial branch nerves [13]. While a detailed and validated approach has been described in the SIS LMBRFN guidelines, a simplified technical concept is provided here (Fig. 3). The angles of fluoroscopic declination suggested by the analysis of CT spine segment sagittal images may provide useful during fluoroscopic imaging to help define the medial branch target. The established fluoroscopic technique defined by SIS utilizes a 35–40° caudal, along with a 15–20° oblique, rotation of the image intensifier to obtain sharp cortical margins at the target sulcus between the SAP and TP where the medial branch is found. Measurement of these angles on radiography for each patient for procedure planning purposes may be unrealistic and time consuming, and therefore these standard angles may be considered. Inaccurate positioning of the image intensifier may result in poor parallel placement of the cannula and minimized efficacy of the lesion along with safety concerns given the proximity to vascular structures and paraspinal musculature. Our proposed angles should be considered as alternative measurements to potentially improve parallel placement of the cannula, however further studies are needed to elucidate the efficacy using this technique.

## 5 Limitations

Due to the smaller sample size of this study and the wide variability in the range of insertion angles, the confidence intervals at each lumbar insertion angle are large and overlapping. Thus, any conclusive remarks on specific differences cannot be made due to the lack of statistical significance. 19 males and 31 females were studied after excluding patients based on our inclusion and exclusion criteria. This could pose a limitation to our analysis with the predominance of females in the study population. Thus, our data was stratified based on sex without any statistically significant differences at any of the lumbar levels. Furthermore, stratifying the data based on BMI and age also did not yield any statistically significant differences in terms of the angle between for the lumbar segments either. These are likely due to the smaller sample sizes from an already small study population to begin with. Of note, the recommendations presented in this paper are only applicable to patients without instrumentation, infection, primary or metastatic disease, compression fractures, kyphoplasty or laminectomies of the lumbar spine as these characteristics can alter anatomy and angle of insertion. It is our hope that the mean angles of insertion presented in this paper serves as proof-of-concept for a larger study to determine standardized angles of insertion for which a population of around 3000 patients is needed for adequate power.

Another limitation in our data collection is that CT scan images are 1.25 ​mm cuts so the exact junction of the SAP and TP may be missed. Additionally, the method utilized to review the images via PACS annotation tool can result in some degree of human variability and error. Each patient’s imaging was reviewed separately by two independent reviewers and using the annotation tools provided in PACS and the cosine formula, the precise angle needed to target the medial branch relative to a starting position perpendicular to a “squared” superior end plate was determined. Prior training of the evaluators on multiple samples and crossed-referencing with each other prior to collecting all the measurements to standardize the method and minimize this human error. Future studies may also consider including more reviewers or use machine learning algorithms to make the measurements to further reduce error and variability. One option would be to 3D print the lumbar spines from the CT images to obtain 3 dimensional views of the junction between the SAP and TP. This may allow for better calculation of the angle needed to place a cannula parallel to the junction.

## 6 Conclusions

This retrospective observational study sought to characterize an angle of cannula insertion at each lumbar level for placing RF cannula parallel to the medial branch nerves in patients undergoing LMBRFN for the treatment of axial low back pain. Mean angles of insertion at each lumbar level were approximately 20° at L1, 20° at L2, 25° at L3, 30° at L4, and 40° at L5 caudally with a starting position perpendicular to a “squared” superior end plate. Future studies with a larger sample size can potentially further elucidate granular differences in insertion angle. Using this knowledge, further investigation can be performed to evaluate the utility of our CT-determined angles when coupled with established fluoroscopic techniques for proper RF cannula placement for the treatment of facet-mediated low back pain.

## Contributors

AG is the guarantor. He developed the idea for the study, designed data collection tools, method of analysis, monitored data collection for the whole study, cleaned and analyzed the data, and assisted in drafting and revising the manuscript prior to submission. AP also assisted in creating data collection tools, performed data collection through imaging review, analyzed the data, assisted in drafting the manuscript, and approved the final version to be published. JC assisted in creating data collection tools, performed data collection through imaging review, analyzed the data, assisted in drafting the manuscript, and approved the final version to be published. PH assisted in creating data collection tools, performed data collection through imaging review, analyzed the data, assisted in drafting the manuscript, and approved the final version to be published. OM assisted in creating data collection tools, performed data collection through imaging review, analyzed the data, assisted in drafting the manuscript, and approved the final version to be published.

## Conflicts of interest

The authors declare no conflicts of interest in relation to this article. Dr. Amitabh Gulati is a consultant for AIS Healthcare, Medtronic, Flowonix, SPR Therapeutics, Nalu Medical, Tremeau Health.

## Funding

This study was supported by the Department of Anesthesiology and Critical Care (NIH Core Grant P30 CA008748).

## Declaration of competing interest

The authors declare the following financial interests/personal relationships which may be considered as potential competing interests:

Dr. Amitabh Gulati reports a relationship with AIS Healthcare, Medtronic, Flowonix, SPR Therapeutics, Nalu Medical, Tremeau Health that includes: consulting or advisory.

## Figures and Tables

**Table:** 

		Combined	Left	Right	Male Combined	Female Combined	BMI<25 Combined	BMI 25–30 Combined	BMI>30 Combined	Age<70 Combined	Age>70 Combined
L1	Mean Angle	20.14654274	20.40115523	19.89193025	19.38369382	20.61409531	20.22627855	20.41632696	20.19865939	19.82979762	20.41636266
	STD	9.260257545	9.488404339	9.115518087	9.038090796	9.436062025	11.87259217	6.736195457	6.55331742	10.74629023	7.871868083
	Margin of Error	1.815010479	2.6300515	2.526692704	2.873696989	2.348826908	3.679354434	2.495121715	2.519014118	3.105529944	2.099602103
	Upper bound	21.96155322	23.03120673	22.41862295	22.25739081	22.96292222	23.90563299	22.91144867	22.71767351	22.93532756	22.51596476
	Lower Bound	18.33153226	17.77110373	17.36523754	16.50999683	18.2652684	16.54692412	17.92120524	17.67964528	16.72426767	18.31676056
L2	Mean Angle	20.94566003	21.2728525	20.61846755	23.14753734	19.59612232	20.35987633	22.22806869	18.16581462	20.48328777	21.33953269
	STD	10.58491433	10.57185885	10.69507751	9.077467364	11.26872487	13.51568896	6.215046202	8.073890977	10.44184518	10.78725949
	Margin of Error	2.074643209	2.930369768	2.964524238	2.886216926	2.805013801	4.188555403	2.302085329	3.103503776	3.017549516	2.87720176
	Upper bound	23.02030324	24.20322227	23.58299179	26.03375426	22.40113612	24.54843174	24.53015402	21.2693184	23.50083729	24.21673445
	Lower Bound	18.87101682	18.34248273	17.65394331	20.26132041	16.79110852	16.17132093	19.92598337	15.06231085	17.46573825	18.46233093
L3	Mean Angle	25.53929558	25.10761049	25.97098067	26.10960401	25.1897517	24.66493267	27.55390324	23.93376294	25.25989918	25.77729992
	STD	8.963005119	8.762826428	9.227295566	7.938629846	9.582658184	11.01812696	8.140811583	5.911654093	9.807849366	8.262023766
	Margin of Error	1.756749003	2.428931564	2.557675841	2.524118998	2.385317661	3.41455292	3.015398809	2.272366676	2.834333453	2.203665291
	Upper bound	27.29604458	27.53654205	28.52865651	28.63372301	27.57506936	28.07948559	30.56930205	26.20612962	28.09423263	27.98096521
	Lower Bound	23.78254657	22.67867893	23.41330483	23.58548501	22.80443404	21.25037975	24.53850443	21.66139627	22.42556572	23.57363463
L4	Mean Angle	31.00591084	32.33083704	29.68098465	30.8936141	31.07473788	30.1178755	31.73635287	29.29621253	29.5181271	32.27328218
	STD	9.338720534	9.829090777	8.718354119	10.18513432	8.865958063	8.276989497	10.40440529	9.01814918	9.424387602	9.161591583
	Margin of Error	1.830389225	2.724484962	2.416604469	3.238404049	2.206916488	2.56506562	3.853845653	3.466464944	2.723518281	2.443600013
	Upper bound	32.83630007	35.055322	32.09758911	34.13201815	33.28165437	32.68294112	35.59019852	32.76267748	32.24164538	34.7168822
	Lower Bound	29.17552162	29.60635208	27.26438018	27.65521006	28.86782139	27.55280988	27.88250722	25.82974759	26.79460881	29.82968217
L5	Mean Angle	40.74196142	40.82916782	40.65475502	39.4759371	41.51791181	39.61173336	39.54163723	41.24365438	41.01080812	40.51294386
	STD	9.486115766	9.255950816	9.804136669	9.57505302	9.424588474	10.70155306	7.557086244	8.323744354	10.12280541	8.998209734
	Margin of Error	1.85927869	2.56561867	2.717568037	3.044426268	2.345970909	3.316445653	2.799183917	3.199544322	2.925351414	2.400022444
	Upper bound	42.60124011	43.39478649	43.37232306	42.52036337	43.86388272	42.92817901	42.34082115	44.4431987	43.93615954	42.9129663
	Lower Bound	38.88268273	38.26354914	37.93718699	36.43151083	39.1719409	36.2952877	36.74245332	38.04411006	38.08545671	38.11292141
